# The Cytotoxic Necrotizing Factors (CNFs)—A Family of Rho GTPase-Activating Bacterial Exotoxins

**DOI:** 10.3390/toxins13120901

**Published:** 2021-12-15

**Authors:** Paweena Chaoprasid, Petra Dersch

**Affiliations:** 1 Institute of Infectiology, Center for Molecular Biology of Inflammation (ZMBE), Medical Faculty Münster, University of Münster, 48149 Münster, Germany; chaopras@uni-muenster.de; 2 German Center for Infection Research (DZIF), Institute of Infectiology, University of Münster, 48149 Münster, Germany

**Keywords:** cytotoxic necrotizing factors, CNF, *E. coli*, *Yersinia*, Rho-GTPases, inflammation

## Abstract

The cytotoxic necrotizing factors (CNFs) are a family of Rho GTPase-activating single-chain exotoxins that are produced by several Gram-negative pathogenic bacteria. Due to the pleiotropic activities of the targeted Rho GTPases, the CNFs trigger multiple signaling pathways and host cell processes with diverse functional consequences. They influence cytokinesis, tissue integrity, cell barriers, and cell death, as well as the induction of inflammatory and immune cell responses. This has an enormous influence on host–pathogen interactions and the severity of the infection. The present review provides a comprehensive insight into our current knowledge of the modular structure, cell entry mechanisms, and the mode of action of this class of toxins, and describes their influence on the cell, tissue/organ, and systems levels. In addition to their toxic functions, possibilities for their use as drug delivery tool and for therapeutic applications against important illnesses, including nervous system diseases and cancer, have also been identified and are discussed.

## 1. Introduction

Many bacterial exotoxins are highly detrimental virulence factors that cause severe cell and tissue damage, and death of animal hosts. These toxins generally manipulate host cell factors that participate in fundamental cellular processes. Several bacterial toxins target small GTPases of the Rho family, which act as molecular switches involved in many signaling pathways implicated in the regulation of actin structures, immune responses, endocytosis, transcription, and cell cycle progression [1,2,3,4]. Both Rho GTPase-deactivating and -activating toxins are known [5].

The family of cytotoxic necrotizing factors (CNFs) are single-chain AB-type toxins, which belong to the class of Rho GTPase-activating toxins. The toxins are made up of 1014 amino acids with a molecular mass of about 115 kDa [6]. The first CNF was discovered in *E. coli* isolated from stool specimens of children with acute gastroenteritis and severe diarrhea. This factor showed a necrotizing effect on rabbit skin, induced striking morphological alterations on HeLa cells, and caused cytotoxicity by inhibiting cell division. Approximately 48 h after application, the great majority of treated cells became multinucleated and 4–5 times larger than the controls [7,8]. Therefore, this protein toxin was named cytotoxic necrotizing factor 1 (CNF1) [7]. Today, it is well known that *cnf1*-positive intestinal *E. coli* isolates belong to the pathogen phylogroups B2 and D with a low prevalence ranging from 8–30% in the human gut microbiota [9]. In contrast, CNF1 production is a common property among pathogenic extraintestinal *E. coli* strains (30–50%), i.e., in uropathogenic *E. coli* (UPEC), causing urinary tract infections (UTI), especially in *E. coli* responsible for prostatitis [10]. In addition, the *cnf1* gene is present in isolates associated with neonatal meningitis [11,12].

At a later date, three other highly related types of CNFs were identified in human and animal clinical isolates of enteropathogenic *E. coli* and *Yersinia pseudotuberculosis* [13,14,15,16]. CNF2 is 86% identical to CNF1 and was found to be encoded by a transmissible Vir plasmid of invasive *E. coli* strains isolated from calves. CNF2-mediated cell alterations were characterized by a lower degree of multinucleation of HeLa cells compared to CNF1, but bacterial extracts containing CNF2 seemed more potent, causing necrosis in rabbit skin [13]. Initially observed CNF-mediated actin rearrangements in epithelial cells indicated the small GTP-binding proteins of the Rho family as possible intracellular targets. Using radiolabeled RhoA, Oswald et al. could demonstrate that RhoA was shifted to a higher molecular weight after treatment with the CNF2 toxin, indicating a role of CNF2 in the modification of Rho proteins [17]. Later, two groups simultaneously showed that the catalytic region of CNF toxins covalently modifies small Rho GTPases through the site-specific deamidation of a glutamine (Gln63) to glutamate (Glu63) in the active site (switch II region) of RhoA or Gln61 of Rac1 and Cdc42 [18,19]. These alterations lock these key regulators in their active states and cause a multitude of downstream effects, leading to massive cell and tissue damage and the development of severe acute disease symptoms in infected animal hosts.

In 2007, a third member of the CNF family was discovered in necrotoxic *E. coli* (NTEC) from sheep and goats, and in a strain of *Shigella boydii* [20,21]. It shares about 70% sequence identity with CNF1 and CNF2. As reported for CNF1 and CNF2, CNF3 deamidated all three Rho GTPases RhoA, Rac1, and Cdc41. However, in contrast to CNF1, this variant activated RhoA 5–10 times stronger and over a longer time period in HeLa cells compared to CNF1. Notably, this feature is very similar to CNF_Y_, a CNF variant of *Yersiniae* [21,22].

**Figure 1:**
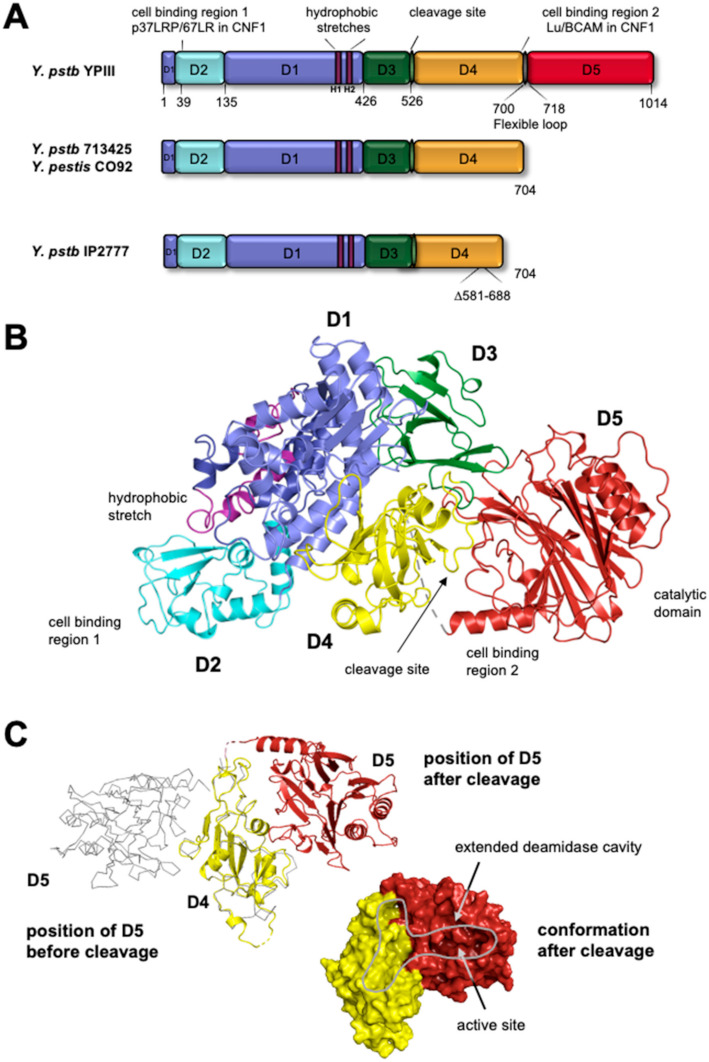
Domain structure and organization of CNF_Y_ and its derivatives. (**A**) Sequence comparison of the *cnfY* gene from the *Y. pseudotuberculosis* strains YPIII, 713425, and IP2777, and *Y. pestis* strain CO92. The C-terminal and internal deletions of the *cnfY* derivates are indicated. The amino acid number of the domain ends according to the amino acid sequence are given. D1-D5 illustrate the individual domains. (**B**) Representation of CNF_Y_ crystal structure colored according to the domain boundaries shown in (**A**). Domain D1 (dark purple) includes the p37LRP/67LR receptor-binding motif with predicted membrane-inserting a-helices (light purple); linker domain between D4–5 contains the second binding site. The cleavage site, which is used to separate D4–5 from the full-length CNF_Y_ protein upon the release into the cytoplasm is indicated. (**C**) Representation of the two conformations of the D4–5, in the full-length and the cleaved state. The active deamidase site exposed in the cleaved derivative is indicated by an arrow.

The homologous chromosomal gene (*cnfY*) of 3045 bp encoding CNF_Y_ has been identified in *Y. pseudotuberculosis* strains YPIII and IP2666 of serogroup III. The amino acid sequence of CNF_Y_ is approximately 65% identical to CNF1 [14], and the encoded protein was found to activate Rho GTPases and induce multinucleation and the development of giant cells, which is very similar to *E. coli* CNF1 [14,23,24,25]. Flanking sequences had a different GC content and had significant similarities to transposases, indicating that *cnfY* was acquired by horizontal gene transfer from other bacteria, e.g., *E. coli*. Interestingly, many other *Y. pseudotuberculosis* and all *Y. pestis* strains contain a deletion of the 3′ end of the *cnfY* gene, eliminating all sequences encoding the catalytic region (Figure 1A) after nucleotide 2114 (704 aa). Several *Y. pseudotuberculosis* strains also carry a second deletion between 1745–2065 (581 aa-688 aa) (see Figure 1A).

Additional homologs with about 62–65% protein sequence identity have been discovered in genomes of *Salmonella enterica serovar* Loma Linda, *Photobacterium damselae* (CNFp), and *Moritella viscosa* (CNFm), and the exchangeability of the active domains of the CNF-like toxins has been demonstrated [26,27]. Moreover, several functional CNF-like domains have been identified in other toxins or bacterial effectors. For instance, the *cnf* genes showed approximately 50–60% identity in a 613 bp overlap (between codon 200 to 420) with a gene (*toxA*) encoding the large dermonecrotic toxin PMT of *Pasteurella multocida* (146 kDa) [6,28]. This factor is responsible for turbinate atrophy in progressive anthropic rhinitis in pigs [29]. In addition, the dermonecrotic toxin (DNT) of *Bordetella bronchiseptica* and *B. pertussis*, which has shown to directly target Rho GTPases [28,30,31], shares homology with the CNFs in the C-terminal catalytic domains (residues 1136–1451 of DNT, 709–1014 of CNF1, and CNF_Y_).

In fact, the homologous regions within the C-terminus contain conserved amino acids, including cysteine C866 and histidine H881 in the CNFs, which have been shown to be essential for enzyme activity [24,32]. Similarly, amino acid residues C1165 and H1205 are crucial for the toxicity of *Pasteurella multocida* toxin (PMT) [33,34,35]. The toxins also share the catalytic dyad of conserved cysteine and histidine residues with eukaryotic transaminases and cysteine proteases. Apart from deamidation, transglutamination activities were reported for CNF1 [36,37]. Notably, CNF-like activity domains have also been discovered in bacterial effectors, e.g., the type III secreted factor VopC of *Vibrio parahaemolyticus*, HopBJ1 of *Pseudomonas syringae*, and a type VI secreted effector Vpr01570 of *Vibrio proteolyticus* [27]. The wide range of bacterial genomes containing CNF-like activity domains indicate an extensive horizontal gene transfer of the modules.

## 2. Regulation of *cnf* Gene Expression

The *cnf1* gene is located within the pathogenicity island II downstream of the *hlyCABD* operon [11], and their expression is linked by the transcriptional antiterminator protein RfaH [38]. Using different *cnf1-lacZ* translational fusions, Fabbri et al. [39] demonstrated that the first 48 codons of *cnf1* are involved in the translational regulation of CNF1 synthesis, most likely through a ribosome-binding site complementary sequence. Moreover, it has been reported that *cnf1* is downregulated by the histone-like nucleoid structuring protein (H-NS) [40].

A first study addressing the expression of the *cnfY* gene in *Y. pseudotuberculosis* revealed that this toxin gene is only weakly expressed at 25 °C, but strongly induced at body temperature (37 °C) [25]. More detailed studies then showed that the CNF_Y_ synthesis is controlled by an RNA thermometer, which encloses the ribosomal binding site of the *cnfY* mRNA into a double-stranded stem of a hairpin structure, which melts and liberates the ribosomal binding site upon a temperature shift from 25 °C to 37 °C [41,42]. A very high expression of *cnfY* could generally be observed in complex media during stationary phase, resembling conditions found in the mammalian intestinal tract. In agreement with this observation, a very strong bioluminescent signal of a *cnfY-luxCDABE* fusion was detected two days post-infection, demonstrating that the toxin gene is highly expressed during infection [25].

## 3. Structure and Domain Organization of CNFs

All CNFs are single-chain toxins, of which, the N-terminal part is important for the bacterial export and cell entry and the C-terminal part promotes the biological activity, i.e., is responsible and sufficient to deamidate Rho GTPases [43]. To further characterize the enzymatic reaction of the toxins, the crystal structure of the catalytic domain of CNF1 (residues 720–1014) was determined [44]. The 3D structure revealed the active pocket and recognition site for the Rho GTPases. The center of the CNF catalytic domain is highly conserved. It is formed by a β-sandwich, consisting of two mixed β-sheets, which is surrounded by α-helices and five loop regions (loop 2, 6, 7, 8, and 9). Loop 8 (aa 964–970) and loop 9 (aa 996–1003) were found to be crucial for Rho GTPase recognition. They contain a catalytic triad composed of cysteine C866 and histidine H881, located in a deep pocket of the catalytic domain that restricts access of random substrates, which may explain the high specificity of the toxins [44,45].

To achieve an understanding of the toxin export, cell binding, and intracellular transport mechanisms, the crystal structure of the full-length CNF_Y_ toxin was resolved. CNF_Y_ adopts a compact, modular structure of five distinct domains (D1-D5), with the domain residues 1–22/135–424 (D1), 23–134 (D2), 425–529 (D3), 530–700 (D4), and 718–1014 (D5) (Figure 1A,B) [46]. The boundaries and molecular functions of the individual domains are conserved as modular domain swapping among the CNF family remains their functionality [26]. The CNF_Y_ domain D1 is built by two amino acid stretches of the N-terminus, separated by D2, and forms a compact bundle of α-helices. Residues 387–412 of D1 form a loop, with two hydrophobic, transmembrane helices (H1 and H2) that play a vital role for the insertion of the toxin into the endosomal membrane [47] (see Intracellular Uptake and Trafficking). Domain D2 protrudes from D1 and contains several surface-exposed loops, one of which (residues 53–75) represents a host cell receptor binding region (see Receptor Binding of CNFs). The central D3 domain of CNF_Y_ (residues 426–526) forms an imperfect β-barrel composed of six anti-parallel β-strands. D3 is connected to domain D4 (residues 527–700) via a linker that is partially shielded by the C-terminal catalytic domain D5, comprised of seven β-sheets and the conserved dyad active site (C866/H881) [46].

## 4. Export of CNFs

Several AB toxins are exported via T2SS, such as the cholera toxin (CT) of *Vibrio cholerae* and the heat-labile toxin (LT) of enterotoxigenic *Escherichia coli* (ETEC) [48,49]. The subunits A and B containing the N-terminal signal peptide are synthesized in the bacterial cytosol and released via the Sec-pathway. Thereafter, the subunits A and B are assembled into the functional AB_5_ toxin, and the holotoxin is transported across the bacterial membrane via a T2SS [50,51].

In contrast, very little is known about the export mechanism of CNFs from bacteria. Both CNF1 and CNF_Y_ have been identified as exotoxins, but they do not contain a typical consensus signal peptide [11,12,14]. Therefore, T2SS does not seem to be involved in the export systems of those toxins.

Active secreted CNF_Y_ was found in the supernatant of cultured *Y. pseudotuberculosis*, whereas *E. coli* CNF1 seemed to be more membrane-associated (>95%) [40,52]. Ferredoxin seems to be essential for the secretion of CNF1 across the bacterial inner membrane and the YgfZ protein appears to assist crossing the outer membrane from the periplasm [53,54]. Recently, it was also postulated that CNF1 and CNF_Y_ are directly released and/or exported from the bacteria to the surrounding environment via tight association to outer membrane vesicles (OMVs) [52,55,56]. It was concluded that the CNFs are localized inside the vesicles and, thus, are protected from external adverse conditions [40,52]. However, application of proteases demonstrated that CNF1 is mainly present on the exterior of the OMV [55]. Accordingly, the majority of the CNF_Y_ toxin remained in the supernatant after the OMVs were separated from a *Y. pseudotuberculosis* culture by ultracentrifugation (P. Chaoprasid, unpublished results). CNF1 and CNF_Y_ bound to OMVs are active and have been found to intoxicate host cells [52,55], suggesting that the CNFs can be transmitted as secreted protein or via binding to OMVs. However, OMVs carrying CNF_Y_- or CNF1-treated cells have been found to exert a considerably delayed activation of Rho GTPases and CNF-triggered phenotypes [57] (Chaoprasid unpublished results). It is possible that cell binding is reduced, or free and OMV-bound CNF take different routes to enter target cells.

The analysis of CNF_Y_ variants with deletions of one or more domains revealed that the first three domains (D1–3) are important for toxin secretion and act as an export and translocation device for the catalytic unit (D4–5). Integration of structural and functional data suggest that domain D1 constitutes a membrane-spanning transport unit, which promotes bacterial export and exposes domain D2, harboring a cell recognition site to trigger endosomal uptake [46].

## 5. Receptor Binding of CNF Toxins

Over the past decade, most studies have reported that AB toxins contain one receptor binding region, either at the N- or C-terminal region based on their structures. For example, the binding domains of diphtheria toxin (DT) of *Corynebacterium diphtheriae* and exotoxin A (ExoA) of *Pseudomonas aeruginosa* are located at the N-terminal and C-terminal domain, respectively [58,59]. In addition, the CNF-related *P. multocida* toxin, PMT, comprises one receptor binding domain between amino acid residues 1–580 [60]. In contrast, the CNF-type toxins were found to interact with at least two distinct receptors [61].

**Figure 2:**
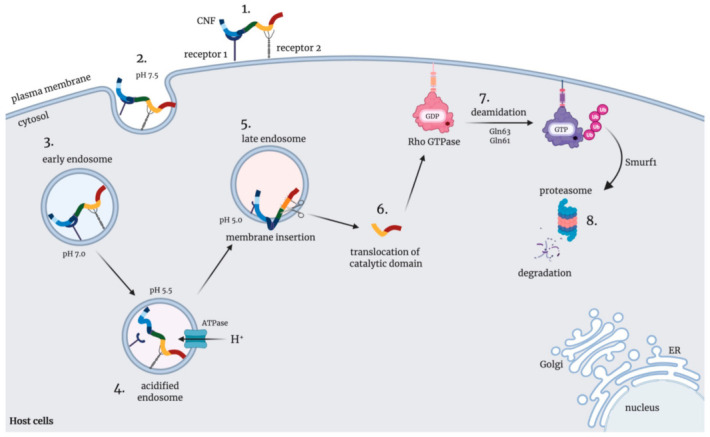
Uptake and intracellular trafficking of CNFs. The CNFs are secreted directly and/or bound on outer membrane vesicles. (1) They interact with two different cell receptors with the N-terminal and C-terminal domains, and (2) internalize into host cells by endocytosis and are found in the early endosomes (3). Acidification of the late endosome induces a conformational change (4) and proteolytic processing (5), releasing the catalytic fragment D4–5 into the cytosol (6). The D4–5 protein fragment deamidates Rho GTPases at the cell membrane (7), which triggers multiple cell responses, including ubiquitinylation and proteolytic degradation (8).

CNF1 was first reported to internalize into host cells via host cell receptor-mediated endocytosis by the interaction of the first 190 N-terminal residues. In particular the region between amino acid 53 to 75 was found to play an essential role in CNF1 host cell receptor binding [39] (Figure 1 and Figure 2). Later, the 37-kDa non-integrin laminin receptor precursor protein (37LRP), involved in cell migration and adhesion, was identified as a specific receptor for CNF1 and CNF2 [62].

The analysis of truncated CNF1 mutants further revealed that an additional cell binding site is present in CNF1, which is located within the catalytical region (D4-D5) [61], and this was also reported for CNF_Y_ [46] (Figure 1 and Figure 2). In line with this, the Lutheran adhesion glycoprotein/basal cell adhesion molecule (Lu/BCAM) was discovered as an additional receptor for CNF1, and the Ig-like domain 2 of Lu/BCAM was identified as a main interaction site [63,64]. Lu/BCAM is expressed in epithelial and endothelial cells and, similarly to LRP, interacts with laminin. The interaction between CNF1 and Lu/BCAM is located at the linker and the beginning of the catalytic domain, most likely between amino acids 683 to 730. As deletions or blockages of each of the receptor binding sites inhibits toxin function, it is assumed that both receptors are required for efficient CNF1 intoxication [61,65].

In contrast to CNF1, competition studies, e.g., pretreatment of the HeLa cells with inactive CNF_Y_, did not inhibit and only delayed uptake of CNF1, and vice versa [64]. Moreover, CNF1 colocalizes with the Lu/BCAM receptor, but not CNF_Y_ [65]. This indicates that the two toxins bind to different cell receptors, probably with overlapping structures. In another study, cells were preincubated with an excess of either inactive CNF3 or CNF_Y_ before intoxication with CNF1. This elucidated that both proteins could block CNF1 intoxication [21]. Moreover, CNF3 and CNF_Y_ exhibit similar cell binding properties//specificities, indicating that they may recognize similar receptors [21]. However, the exact areas required for the binding of CNF_Y_ or CNF3 remain unknown.

As HeLa cell treatment with sodium chlorate, which inhibits the synthesis of heparan sulfate proteoglycans (HSPGs), strongly retarded CNF1 and CNF_Y_ uptake, it was suggested that HSPs are involved in the internalization of both toxins [64]. In fact, glycosaminoglycans (GAGs) were reported to be involved in recruiting CNF_Y_ to the surface host cells [66]. GAGs are large linear polysaccharides, such as heparin (HP), heparan sulfate (HS), dermatan sulfate (DS), and keratan sulfate (KS) [67], that bind to the core proteoglycan (PG) and are attached in close proximity to the extracellular matrix or cell surface proteins. Furthermore, Kowarschik et al. demonstrated that the C-terminal domain (residues 704–1014) of CNF_Y_ binds with high affinity to heparin and heparan sulfate, which is required for intoxication within the endosome [66]. It was established that HSPG plays a role in the uptake of polyarginine, polylysine, and arginine–lysine hybrid peptides [68]. Interestingly, ^772^VKKTKF^779^, a typical heparin-binding motif that is rich in lysine, appears to be essential for the interaction of CNF_Y_ with heparin. Consistent with these data, Chaoprasid et al. demonstrated that the CNF_Y_ D4–5 alone, including the GAG binding site, was sufficient to promote host cell binding [46]. Due to the fact that this C-terminal fragment was more potent for GAG binding, it was postulated that the binding site may be sterically hidden in the full-length toxin, but could be exposed by a conformational change following cell contact via the N-terminal cell receptor binding site [66].

## 6. The Intracellular Uptake and Trafficking

Most bacterial toxins are delivered into host cells via receptor-mediated endocytosis after binding to specific host surface receptors [69,70]. After internalization, toxins are transported to the early endosomes (Figure 2), which serve as sorting platforms for internalized cargo.

Subsequently, the early endosomes are maturated into the late endosomes. Late endosomes fuse with lysosomes and degrade their cargo or recycle the cargo back to the cell surface [71]. To reach the cytosol, toxins need to escape from endosomes during trafficking. For this purpose, they exploit changes in the endosome, such as the acidification and/or the activation of proteases [72,73,74]. On the other hand, bacterial toxins can be retroactively transported from the endosome, through the Golgi apparatus, to the endoplasmatic reticulum (ER) and translocated from the ER to the cytosol [75]. Some toxins (e.g., Shiga toxins, ricin and cholera toxin) can internalize into host cells via more than one mechanism, i.e., via either clathrin-dependent or clathrin- and caveolae-independent pathways, and are subsequently transported through the Golgi to the ER [76,77,78].

The CNFs are taken up into the host cells via a clathrin- and caveolae/lipid raft-independent endocytic mechanism and require an acidic-dependent membrane translocation step to transfer its enzymatic activity into the host cell cytosol (Figure 2) [64,79]. A, an inhibitor of vesicle formation and protein trafficking between the ER and the Golgi apparatus, influenced neither CNF1 nor CNF_Y_ intoxication [79], indicating that CNFs are not transported into the cytosol via the trans-Golgi-network—ER route. In fact, treatment with methylamine, a membrane-permanent weak base known to inhibit lysosomal processing [80,81,82], or nocodazole, a substance inhibiting the transport from early to late endosomes, blocked CNF1 activity, indicating that the CNFs must reach a late endosomal compartment to deliver their catalytic domain into the cytosol [64,79] (Figure 2). Colocalization of CNF_Y_ of *Y. pseudotuberculosis* with early and late endosomal markers in human epithelial cells confirmed this assumption [46].

Subsequent escape from the endosomes is critical for many toxins to exhibit their enzymatic function [83]. In most cases, acidification of the endosome triggers their release into the cytosol [84,85]. Inhibition of acidification via blocking of the endosomal proton pump by bafilomycin A1, monensin, [86], or weak bases (e.g., methylamine, ammonium chloride) completely abolished the intoxication of the CNFs [17,79]. Interestingly, CNF1 and CNF_Y_ can both be directly pulsed through the plasma membrane from the medium into the cell cytosol by external acidification [64]. Based on these observations, it is assumed that the CNF family is transported into the host cells via endocytosis and requires the acidified endosome to deliver catalytic domain to the cytosolic target site [79,87,88,89,90] (Figure 2). C-terminal truncations of CNF_Y_ clearly demonstrated that deletion variants, consisting of domains D1–3, but not of D1–2, were able to reach the cytosol, implying that domain D3 is required for the translocation and endosomal escape process [46]. Surprisingly, CNF_Y_ was still able to reach the late endosome, even when parts of the N-terminal domains were missing (CNF_Y_Δ39–426), but these variants were unable to escape from the endosomal compartment [46,66].

## 7. Release of Activated CNFs into the Host Cell Cytoplasm

The crystal structure of CNF_Y_ revealed potential transmembrane helices within domain D1 between residues 340–368 (H1) and 393–408 (H2), and two conserved glutamic acidic residues 382 and 383 are located within the hydrophilic loop that links both helices (Figure 1) [46]. The substitution of both glutamic acidic residues for positively charged residues eliminated the toxicity of the CNFs [91], supporting a critical role in endosomal escape. Protonation of the glutamic acid residues upon acidification of the late endosome may promote a conformational change that allows insertion of the hydrophobic helices into the endosomal membrane and initiates the translocation process of the toxin [92].

Transfer of the catalytic unit of the CNFs across the membrane can be directly triggered by a low-pH-driven mechanism, which seems to require proteolytic cleavage between residues 532 and 544 by one or several endosomal protease(s), and a 55 kDa fragment, D4-D5, harboring the catalytically active domain is released into the cytosol [93]. Generation of multiple mutations in the potential cleavage region resulted in the identification of the CNF1 (P536A/V537G) variant, which was not cleaved. CNF_Y_ contains conserved amino acids in the same region (residues ^535^IPVYFIDKPYS^545^). However, the triple mutation of CNF_Y_ I535/P536/V537G did not alter translocation and deamidase activity, whereas additional substitutions of amino acids between 538–542 did [46].

Acidification of the late endosome could also activate heparinase, which cleaves heparan sulfate chain from HSPG within the endosome [94]. As CNF_Y_ was found to interact with HSPGs, heparinase activation could promote cleavage and release of the catalytic unit D4–5, as suggested by a previous study [66]. Only CNF_Y_ but not CNF1 binds to HSPGs, yet the release of the catalytic unit was observed for both toxins, indicating a conserved/common release mechanism.

The comparison of the crystal structure of the cleaved, catalytically active CNF_Y_ D4-D5 fragment with the full-length CNF_Y_ toxin revealed that domain D4 changed its position with respect to D5, which liberated D5 from the semi-locked conformation seen in the full-length toxin (Figure 1C). This conformational change exposed the catalytic pocket, leading to a higher deamidation activity of the toxin [46].

## 8. Mode of Action and Pathogenesis

### 8.1. CNF-Mediated Activation of Rho GTPases

All identified CNF toxins, as well as PMT of *P. multocida* and DNT of Bordetella, are AB toxins [24,87,95], which activate the Rho family proteins through site-specific deamidation of glutamine (Glu61/63) to glutamic acid (Gln) (i.e., Glu63 in RhoA and Glu61 in Cdc42 and Rac1) [19,28,96]. The deamidated Rho GTPases have lost the ability to hydrolyze GTP (intrinsic and GAP-stimulated), and, given that they are permanently bound to GTP, are considered active. Notably, the catalytic domain D5 also contains a region that shows homology with the NAD-binding region, however, early analyses with CNF2 and recent analysis with purified CNF_Y_ did not reveal NAD^+^ or ATP binding, i.e., excluding NAD-dependent ADP-ribosylation or ATP-dependent phosphorylation of Rho GTPases [17,46].

Constitutive activation of Rho GTPases by the CNFs induces actin polymerization and actin fiber rearrangements and culminates in the formation of thick actin bundles or complex actin networks, stress fibers, lamellipodia, membrane ruffles, microspikes, and filopodia in human epithelial cells [30,97]. With increased time of exposure to the toxin, the number of microvilli decreases and membrane ruffling develops on the cell surface. Cytoskeletal rearrangements attributed to CNF also impair cytokinesis, but not replication, during ongoing cell cycle progression, resulting in multinucleated cells and an improvement of cell spreading [98,99]. Usually, about 96% of human epithelial cells are mononucleated and only about 4% have two or more nuclei. By contrast, most CNF1- and CNF_Y_-treated cells have two (50–60%), three, or more nuclei (30–40%) [25,100].

Notably, the distinct CNFs appear to show different preferences for the Rho GTPases in vivo [18,101,102,103]. CNF1 seemed to provoke modification of all three Rho GTPases, whereby Cdc42 and Rac1 were somewhat less efficiently altered and RhoA deamidations were only transient in HeLa cells [18,101,102,103]. Hoffmann et al., 2004, and Baumer et al., 2008, both reported that CNF_Y_ is a strong and selective activator of RhoA and did not seem to activate Rac and Cdc42 [23,104]. However, in vitro experiments using purified recombinant CNF proteins showed that all three Rho GTPases were simultaneously activated, and comparative analysis illustrated that the ratio of Rho GTPase activation varies significantly between different cell types [23,105]. Differences in the substrate specificity of the enzymes [23,24,25], cell receptor repertoire [64,105], intoxication conditions (OMV versus secreted toxin, different concentrations and exposure times), and extent and time-course of ubiquitinylation and proteasomal degradation [105] are possible causes for these discrepancies.

### 8.2. CNF-Induced Signaling Pathways

**Figure 3:**
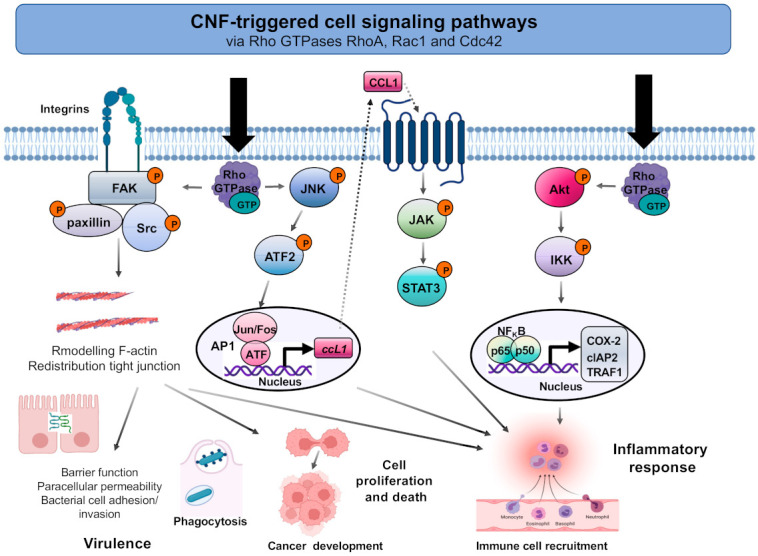
CNF1-induced signaling pathways and host cell processes. Overview of the CNF1-triggered signaling pathways through the activation of the small Rho GTPases RhoA, Rac1, and Cdc42, leading to (I) actin cytoskeleton rearrangements and modulation of cell barriers, (II) interferences with cytokinesis and cell death programs, and (III) modulation of proinflammatory cytokines.

Multiple studies demonstrated that the CNFs trigger/manipulate numerous signaling pathways that are involved in crucial cell processes, such as the regulation of actin structures, immune responses, endocytosis, migration, cell cycles, and control of transcription (Figure 3).

As an immediate response of CNF1-treated human epithelial cells (after 1–2 h), c-Jun N-terminal kinase (JNK), a well-known Rac1 and Cdc42 target, is activated [103]. CNF1 was further reported to stimulate tyrosine phosphorylation of focal adhesion kinase (FAK) and paxillin in Swiss 3T3 cells, with a maximum at 6–8 h after treatment [31]. This was associated with the formation of actin stress fibers and focal adhesion assembly, occurring in a Rho GTPase-dependent manner. In agreement, increase of FAK Tyr^397^ autophosphorylation, which facilitates association of the Src kinase, has been observed, and this was shown to occur by protein kinases of the p160/ROCK family [106]. Subsequent analysis revealed that CNF1 does not seem to stimulate tyrosine phosphorylation of FAK and paxillin through phospholipase C and the PKC-dependent pathway, thus generating Ins(1,4,5)P3 and diacylglycerol (DAG), and does not induce Ca^2+^ mobilization [31]. Moreover, no changes of PtdIns(4,5)P_2_, PtdIns(3,4)P_2_, or PtdIns(3,4,5)P_3_ levels were found increased in CNF1-treated cells, nor were cellular effects of the toxin blocked by the inhibitor LY294002 [101], indicating that CNF1 does not trigger its effect on the actin cytoskeleton via modulation of phosphoinositide (PI)3-kinase activity. In a later study, it was also investigated whether expression of certain antigens involved in cell adhesion are modulated by CNF1. In fact, CNF1 caused a significant increase in the expression of E-cadherin, α/β-catenin, FAK, and the α5, α6, α_v_ chain of integrins, whereas ICAM-1 was downregulated, indicating that both cell–cell and cell–substrate interactions can be manipulated by CNF1 [100].

Another study also demonstrated that CNF1 of *E. coli* strongly induces the expression of the cyclooxygenase-2 gene [107]. COX-2 is known to modulate expression of cytokines, suppress apoptosis, interfere with cell cycle progression, and induce tumor growth, and it could potentially contribute to urothelial carcinomas (for more details see below).

Using different CNFs, Reipschläger et al., 2012, demonstrated that CNF3 and CNF_Y_, but not CNF1, trigger STAT3-dependent transcription in an auto/paracrine manner [22]. This implicated RhoA-GTP-mediated activation of the direct effector ROCK and the MKK1/4/7-JIP-JNK complex in leading to the activation of AP1 transcription factor in human embryonic kidney cells. AP1-triggered transcription of CCL1 stimulates CCR8 receptor-associated JAK activation, which phosphorylates STAT3. The selectivity was explained by a stronger and longer-lasting activation of RhoA by CNF3 and CNF_Y_ [22]. However, as activation of the Rho GTPases Rac1 and Cdc42 by CNFs has also been observed in other cell types and Rac1 and Cdc42 were previously shown to lead to STAT activation via NF_k_B [108], it is also possible that CNF-activation of these Rho GTPases also contribute to STAT activation in other cell types.

CNF1 further influences the activity of activated Rho GTPases by hijacking the host cell proteasomal machinery. CNF1-induced activation of the Rho GTPases was found to be transient, as the deamidated form of the enzymes, in particular of Rac1, promotes an increased susceptibility to ubiquitin/proteasome-mediated degradation [109,110]. About threefold greater appearance of ubiquitinylated forms of Rac1 were detected upon CNF1 treatment, which resulted in a complete loss of the GTPase after 24 h. Moreover, association with ubiquitin-binding proteins could be promoted. The Hace1-HECT E3 ligase ubiquitylates the CNF1-triggered activated GTP-bound form of Rac1, leading to Rac1 proteasomal degradation [111]. Differential sensitivity to the ubiquitin proteasome machinery (Rac1 > RhoA > Cdc42) has been found to result in a dynamic change of actin structures, ranging from strong cell spreading (1–2 h) to intensive lamellipodia formation and membrane ruffing (2–4 h) and to the generation of filopodia-like structures, which is accompanied by an induction of cell motility (6–24 h) [109]. Ubiquitinylation and subsequent depletion of all three forms of activated Rho proteins was shown to require the factor Smurf1 [105]. Notably, CNF1-induced Rho protein depletion is cell line specific, e.g., frequently used cancer cell lines, including HEp2, HEK293, and Vero cells, have a considerably lower Smurf1 expression level and, consequently, a reduced ubiquitinylation capacity of the Rho proteins [105].

Multiple efforts have also been made to characterize CNF-triggered signaling pathways that interfere with cytokinesis, but no typical pathways, such as activation of the MAPKs p42(ERK2) and p44 (ERK1), were triggered by CNF1 [31,112].

Notably, the distinct CNFs appear to show different preferences for the Rho GTPases in vivo, but are likely to stimulate the different Rho GTPases simultaneously. Thereby, they may trigger conflicting signal processes in host cells, which are hardly predictable, as the amounts of the Rho GTPases and the Rho GTPase pathway molecules, as well as the ubiquitin/proteasome components, can differ between different cell types and host origins. In fact, the rates of responsiveness of different cell lines are very different. While HeLa, HEp2, and HEK293 cells responded to CNF1 and CNF_Y_, in contrast, Caco2, HT29, and CHO-K1 were not responsive to the *Yersinia* toxin [64], which could also be due to the absence of the cellular toxin receptor or defects in the uptake machinery.

### 8.3. CNF-Mediated Influence on Tissue Integrity, Cell Barriers, and Cell Death

Activation of Rho GTPases with diverse global functions obviously has major consequences for the functionality of cells, tissues, and the entire organism. To understand the overall influence of these toxins is quite challenging, as the cell specificity of the CNFs is rather broad and their effect varies strongly depending on the molecular cell equipment. Nonetheless, multiple studies exist which have addressed various aspects of the role of CNF toxins and document a plethora of different outcomes.

In vitro studies have shown that the capability of suspended HeLa cells of cell matrix binding is significantly increased upon intoxication with CNF1 and CNF_Y_. This was associated with the formation of more focal adhesions and reduced efficacy of migration compared to untreated cells [113]. This also resulted in pronounced cell spreading upon cell matrix binding with CNF1, but not with CNF_Y_, suggesting that cell spreading is more dependent on Rac1/Cdc42, which is more activated by CNF1. CNF-mediated increase of cell matrix adhesion also decreases the barrier function of intestinal tight junction in a Rho-GTPase-dependent manner, leading to a decrease in transepithelial resistance and an increase in intercellular gaps and paracellular permeability [114,115]. CNF1 seems to trigger remodeling of F-actin structures at the apical surface, which reduces the number and length of microvilli, enhances formation of actin filaments along the junctions and cell–cell contact sites, and enhances generation of stress fibers at the basolateral side [115,116]. This is accompanied with a redistribution of key tight junction proteins involved in epithelial layer function, including Occludin, ZO-1, pMLC, and JAM-1. Occludin seems to be internalized into an calveolin-positive early endosomal compartment upon toxin treatment, through which it can be recycled back to membrane [115]. In contrast to this work, CNF1 was able to decrease the transmigration of polymorphonuclear leukocytes across a T84 monolayer [116,117]. This enhanced adhesion of *E. coli* increased the nutrient supply and reduced the number of attacking neutrophils in the intestinal lumen. A study by Baumer et al. also addressed the influence of CNF1 and CNF_Y_ on endothelial barriers. Their work revealed a cell-type-specific modulation of gap formation and barrier breakdown. For instance, in microvascular myocardial endothelial monolayers of MyEnd cells, CNF1 strengthened barrier properties, whereas in microvascular mesenteric or dermal endothelial monolayers of MEdEnd or HDMEC cells, barrier function was not affected, or, in the case of macrovascular pulmonary artery endothelial cell (PAEC) monolayers, the permeability was increased [104]. CNF-promoted Rac1 and Cdc42 activation seems to be required for barrier maintenance, whereas triggered RhoA function seems to support barrier destabilization. In addition, it has been reported that CNF-1 enhances the adherence of neutrophils to epithelial monolayers, and this paralleled a clustering of β_2_ integrins [118]. Prolonged CNF-1 incubation also caused profound alterations of the epithelial cells, resulting in increased cell motility inside the monolayer and altered cellular junction dynamics [109], consistent with observed enterocolitis [118].

CNF1 was further shown to induce actin rearrangements (membrane ruffling and stress fiber formation) and increase the phagocytic-like activity of human epithelial cells, leading to the digestion of apoptotic cells similar to professional phagocytes [56,119,120]. Similarly, Doye et al. reported that CNF1 allows type I pili-mediated internalization of UPEC via β1-integrins into non-phagocytic uroepithelial cells [109,121]. Moreover, CNF1 promotes invasion of *E. coli* K1 into human BMECs and its capacity to penetrate the blood–brain barrier in the hematogenous meningitis model is significantly reduced [122,123]. Work with CNF1 encoded by UPEC also illustrated that β_1_-integrins are recruited in CNF1-intoxicated cells and seem to be crucial for bacterial cell entry [121]. In this study, they showed that Tollip, an adaptor protein that (i) interacts with ubiquitinylated Rac1 and (ii) binds proteins of the Tom1 family to recruit Tom1-bound clathrin to membranes and regulate interleukin-1 receptor sorting in late endosomes, colocalizes to UPEC at the entry site and participates in the internalization process. In contrast to these studies, CNF1 was found to negatively regulate the CR3 or CD11b-CD18/integrin activation-dependent phagocytic activity of human monocytes and OmpA-mediated invasion of *E. coli* K1 into murine macrophages [124,125]. A later study also reported CNF1-mediated reduction of phagocytosis by human neutrophils, which was accompanied by β_2_ integrin clustering into filopodia [118]. Moreover, CNF1 increased the generation of superoxide/reactive oxygen species (ROS), suggesting that CNF-1 affected the NADPH oxidase machinery and, thereby, cell progression by induction of cyclin D1 expression [118,126].

Several lines of evidence also implicate the CNFs in the control of cell division, and they are thus classified as cyclomodulins [127]. Intoxication of mammalian cells with the CNFs induced the formation of giant mononucleated cells blocked in the G2/M phase while DNA synthesis still occurred [16,25,128,129]. The number of cells in G1 (2n DNA content) was reduced, while the number of cells in G2/M (4n DNA content) increased. A study by Malorni and Fiorentini showed that CNF1 triggers mitotic catastrophe, and this leads to aneuploidy and multinucleation, in line with an anti-apoptotic activity [130]. The first evidence that the CNFs also influence cell death has been provided by a study by Fiorentini et al., in which CNF1 was found to protect human epithelial cells from UV-radiation-induced apoptosis [100]. This work showed that CNF1 induced an increase of the adhesion capability on a substrate (e.g., via integrin ligation), which is important for counteracting apoptosis. Differential expression of surface antigens in human epithelial cells involved in cell adhesion could contribute to this process [100]. Moreover, the presence of Rac1-GTP, as well as inactivation of RhoA by ubiquitinylation following degradation, seems critical for the capacity of CNF1 to promote cell survival [131]. CNF1 also reduces the mitochondrial membrane depolarization induced by UV radiation, induces changes in the mitochondrial (network) morphology, and increases the amount of the anti-apoptotic mitochondrial outer membrane proteins Bcl-2 and Bcl-X_L_ [100,131]. The increase of Bcl-2 mRNA and mitochondrial changes was dependent on the Rac1/PI3 kinase/Akt/IKK/IKBα/NF_K_B pathway, which was shown to be involved in the protection against apoptosis [131]. Moreover, TRAF1 (TNF-receptor associated protein-1) and cIAP2 (mammalian inhibitor of apoptosis protein-1 homologue C), which impair TNF-α receptor cell death signaling, were found to be modulated by CNF1 [112]. Besides its influence on mitochondrial morphology, CNF1 also stimulates mitochondrial activity in intestinal epithelial cells by increasing their ATP production without affecting the equilibrium between FAD generation and oxidation, likely due to an increment of the mitochondrial electron transport chain [132]. This was accompanied by an elongation of the mitochondria, which, together with the increased ATP production, seems to be induced by CNF1-dependent activation of protein kinase A, leading to the phosphorylation of Drp1, controlling mitochondrial fission, which could be the basis for the anti-apoptotic activity of the toxin [132].

However, depending on the concentration and the cell types, CNF1 and CNF_Y_ have also been found to induce apoptosis, e.g., in uroepithelial 5637 cells and in the human androgen-dependent prostate cancer cell line LNCaP, which is frequently used to study prostate cancer [133,134]. Analysis of the underlying mechanism revealed that induction of cell death is associated with loss of plasma membrane asymmetry, PARP cleavage, and depolarization of the mitochondria, and this requires CNF-mediated activation of ROCK and acto-myosin, leading to the activation of caspase-3/7.

Considering the activity of the CNFs on counteractions of cell death, boosting of cellular motility, COX-2 expression, cytokine release, and NF_K_B activation raised the question of whether these toxins can be linked to the development of cancer. Notably, a high prevalence was observed of colonic mucosa-associated *E. coli*-producing CNF1 from patients with colorectal cancer (CRC) [135]. However, it is still unclear whether the CNF1-positive bacteria are the cause for CRC development or whether they replace other bacteria because they are better adapted to the cancer environment. Based on the toxin function, it is assumed that CNF-producing *E. coli* can act as passenger bacteria, which reinforce and favor tumor evolution and progression but do not cause the initiation of the carcinogenic process [135]. In fact, a recent study of the same group reported that CNF1 induces an epithelial to mesenchymal transition in transformed intestinal epithelial cells—a crucial step in malignant tumor conversion and invasiveness—by upregulating the transcription factors ZEB1 and Snail, leading to delocalization of E-cadherin and β-Catenin, and the activation of the serine/threonine kinase mTOR [136]. In contrast, non-transformed epithelial cells needed additional coercing action of inflammation to develop a mesenchymal behavior. In other studies, CNF1 was found to promote cancer development directly. This occurs through the induction of COX-2 expression, activating nuclear factor-kappa B (NF-_k_B) and inhibiting apoptosis [109,131,137]. Another study suggested that CNF1 promotes prostatic cancer progression and metastasis via CDC42-PAK1 signaling pathways and participates in colon cancer development [138]. Moreover, CNF1 promotes bladder cancer angiogenesis by activating RhoC [139]. The link between CNF1 and cancer promotion might be due to the modification of Rho proteins leading to permanent activation of the molecule and to tumor onset [140].

### 8.4. Influence of CNFs on Bacterial Virulence and Host Immune Responses

Based on the influence of CNFs on a wide variety of crucial host cell processes, it is not surprising that these exotoxins have a strong influence on bacterial virulence. A role for virulence was first reported for CNF1 in a murine model for urinary tract infections. More CNF1-positive strains were recovered in higher numbers from the urine, bladder, and kidneys compared to CNF1-deficient strains [117]. Another study using a CNF_Y_-encoded *Y. pseudotuberculosis* strain in an aerosol mouse model revealed that a CNF_Y_-negative strain did not cause lethal disease [141]. In 2013, a complementary study using the natural oral infection route demonstrated that CNF_Y_ is crucial for *Y. pseudotuberculosis* YPIII virulence. Whereas an infection with wildtype succumbed to the infection between day 4 and 6, all mice infected with the isogenic *cnfY*-deficient strain remained alive 14 days post-infection, although comparable numbers of the bacteria were detected in the intestine and underlying lymphoid tissues [25].

Several lines of evidence further implicated the CNFs in pathogenesis of the intestine (e.g., colibacillosis, diarrhea, enteritis, colitis), in particular the induction of detrimental proinflammatory responses. The first report on this topic by Fournout et al. documented a lower pathogenicity of a *cnf1 E. coli* mutant in germ-free piglets compared to wildtype. No difference in the bacterial persistence in the different organs was observed, but inflammatory changes in the ileum and colon were significantly reduced in the absence of CNF1. Subsequent analysis of the production of inflammatory cytokines revealed a lower expression of the proinflammatory cytokines IL-6 and IL-8 in the ileum [142]. Components implicated in the signal transduction from CNF1-activated Rho GTPases to the NF-_K_B pathway triggering cytokine expression are likely to include the receptor-interacting protein kinase 2 (RIPK2) and the nucleotide-binding oligomerization domain protein 1 (NOD1) [143,144]. Another study addressing the course of bacteremia in mice after intravenous infection clearly showed that CNF1 potentiates LPS-triggered secretion of proinflammatory mediators, in particular IL-1β and IL-6. This immune response is abrogated in caspase-1/-11-impaired mice, indicating that these inflammatory caspases play a major role in the clearance of the bacteria from the bloodstream [145]. Moreover, CNF1-mediated RhoA activation antagonizes pyrin inflammasome activation [146].

Similarly, drastic reductions in intestinal inflammation and tissue damage were observed during mouse infection with a *cnfY* mutant of *Y. pseudotuberculosis* compared to the wildtype isolate. This was accompanied with a significantly lower expression of proinflammatory cytokines, in particular IL-1 and IL-6, and, most strikingly, led to a much higher percentage of persistent intestinal infections (10% wildtype versus 70% *cnfY* mutant) [147]. Flow cytometry analysis revealed variations of the immune cell populations, whereby, in particular, the number of neutrophils and macrophages were significantly increased in *cnfY* mutant-infected mice and not reduced as observed in wildtype-infected mice [25,147]. Further analysis revealed that CNF_Y_ of *Y. pseudotuberculosis* enhances the injection of cytotoxic Yop effector proteins into phagocytes to block their phagocytic function and induce cell death [25,148]. A tissue-RNA-Seq analysis of Peyer’s patches and the caecum of CNF_Y_-positive *Y. pseudotuberculosis*-infected mice showed that the inflammation in the Δ*cnfY* mutant-infected mice was much less severe during the acute phase. Moreover, it became evident that the absence of the toxin triggered interferon-γ-mediated protective/tolerogenic immune mechanisms and non-inflammatory antimicrobial activities, allowing the bacteria to escape the efficient elimination strategies of the host and enabling them to remain long-term and asymptomatic within the tissues [147]. The persistent infection was characterized by an overall transcriptome of the colonized lymphoid tissues, which was similar to uninfected mice—only a few transcripts were enriched, indicating that neutrophils and mast cells were recruited during *Yersinia* persistence to counteract the pathogen [147]. Thus, absence of the toxin allows the bacteria to efficiently evade the host immune response and persist long-term in the host without symptoms—very similar to commensals.

The role of CNF1 was further tested in a murine model for urinary tract infections. More CNF1-positive strains were recovered in higher numbers from the urine, bladder, and kidneys compared to CNF1-deficient strains. Moreover, deeper and more extensive inflammation of the bladder and tissue damage were detected in mice infected with CNF1-positive strains, consistent with a role for CNF1 in uropathogenic *E. coli* (UPEC) pathogenesis [149,150]. Based on the knowledge that CNF1 causes epithelial barrier damage, it can be speculated that CNF1-positive UPEC strains may get better access to deeper tissues. In addition, infection of rat prostates with CNF1-positive UPEC strains caused more inflammation-mediated tissue damage, indicating a role of CNF1 in *E. coli* virulence in acute prostatitis [117,139]. In this context, CNF1 was found to enhance neutrophil recruitment and this was accompanied with Rho GTPase-mediated downregulation of the transcription of the scavenger receptor CD36 [150]. To also assess the individual contribution of certain host cells to the overall CNF-triggered response in the distinct pathogenic processes/diseases, the transcription and secretion patterns of proinflammatory cytokines by specific host cells and the influence on host immune cells were analyzed. Falzano et al. 2003 reported that, in particular, TNF-α, IFN-γ, IL-6, and IL-8 increased significantly upon toxin treatment between 3–18 h in uroepithelial cells [126]. A later global transcriptome analysis of primary endothelial cells intoxicated by CNF1 further revealed that the most activated genes formed a similarly coherent set of inflammatory mediators (including MCP-1, MIP-3 α, IL-6, and IL-8) implicated in the recruitment and activation of leukocytes and dendritic cells together with the induction of the leukocyte cell binding receptors, E-selectin and ICAM-1 [112]. A transcriptomic study of the bladder of mice infected with a CNF1-producing UPEC strain confirmed CNF1’s contribution to the expression of genes involved in inflammation, innate immunity, and signal transduction, whereas expression of metabolic and transport-associated genes was reduced [151]. In particular, proinflammatory cytokines and cytokines (including IL-6, IL-17, CXCL1, CXCL-1, TNFα), their receptors and triggered signaling components, and Jak-STAT and Toll-like receptor (TLR) signaling pathways were upregulated. The analysis of bladder sections further revealed a severe pathology: significant edema, neutrophil infiltration, and urothelial damage and hemorrhage [151]. The pathology was similar, although less intensive, when the mice were challenged with CNF1, demonstrating CNF1-triggered inflammatory responses independent of LPS.

Besides its influence on intestinal and urogenital infections, it was reported that CNF1 contributes to *E. coli* meningitis by affecting bacterial invasion of the blood–brain barrier and the penetration into the brain [152]. In a newborn mouse model of meningitis, the toxin exacerbated the disease severity in brains [125].

## 9. Potential Therapeutic Applications

For many years, toxins have been used for medical or cosmetic applications. For example, *Clostridium botulinum* neurotoxin serotypes A and B are commonly used as locally administered cosmetics (Botox) to reduce winkles [153]. Additionally, it has been used as a therapeutic to reduce symptoms of hyperactive muscles, including headaches, urinary incontinence, eye movement abnormality, and limb spasticity [154,155]. The extreme potency, high efficacy, and long-lasting and specific activity on Rho GTPases also suggests strong potential for therapeutic exploitation of the CNF toxin family. In fact, a rapidly increasing number of studies encourage the potential use of CNF1 for the treatment of central nervous system diseases and cancer.

CNF1 has been identified to lead to rearrangements of the cerebral actin cytoskeleton, enhance neurotransmission, and improve associative and spatial learning and memory after activation of cerebral Rho GTPases [132,156,157]. Moreover, CNF1 has been found to (i) correct deficits in reversal learning [158]; (ii) increase ATP levels in the hippocampus and rescue cognitive deficits/special and emotional memory processes [159]; and (iii) improve object recognition, location, and discrimination in mice [160,161]. The effects persisted for weeks and suggested that learning ability, memory performance, and/or Alzheimer’s pathology could be improved through pharmacological manipulation by CNF1 [159]. Follow-up studies revealed that CNF1 enhanced synaptic plasticity and improved learning and memory in mice intoxicated with a single intracerebroventricular injection of CNF1. This influence seems to be mediated by astrocytes. CNF1 increased their supporting activity on neuronal growth and differentiation, likely by the reduction of interleukin 1β levels [132,162]. Injection of CNF1 into the primary visual cortex of naïve adult rats was also found to increase functional plasticity, i.e., spine density and length in pyramidal neurons [163,164]. In line with these findings, CNF1 administration markedly improved the behavioral phenotype of a mouse model of Rett syndrome by reversing the atrophy and the cognitive deficits, suggesting CNF1 as a new treatment for Rett syndrome [165].

The actin rearrangement induced by CNF1 further demonstrates the therapeutic effect on other neurodegenerative disorders, such as Parkinson’s disease, as CNF1 displays neurotrophic effects on dopaminergic neurons [159,166]. Strikingly, a single injection into a mouse model with high susceptibility to epileptic seizures resulted in a remarkable reduction of the seizure phenotype with a significant augmentation in neuroplasticity markers and in cortex mitochondrial ATP content [167]. CNF1 was able to trigger a significant increase in the cellular ATP content and mitochondrial marker proteins in fibroblasts derived from a patient with myoclonic epilepsy with ragged-red fibers (MERRF), pointing at a possible strategy to limit damage in mitochondrial diseases [168].

The senescent-like phenotype of CNF-intoxicated cells (cell enlargement, blockage of cell division, cell immobilization and degeneration), suggests that the CNF family could have some anti-tumoral properties and could be useful to treat cancer. In fact, the activation of the Rho GTPase by CNF1 and CNF_Y_ was shown to induce apoptosis of a human androgen-dependent prostate cancer cell line, LNCaP, derived from a metastatic lesion of human prostatic adenocarcinoma to study prostate cancer [133]. CNF1 treatment further blocked mitosis/cytokinesis, elicited endoreplication and polyploidization in human colon cancer cells, and drove them into senescence [129]. A therapeutic potential also became evident in a study demonstrating that CNF1 is able to block the proliferation of glioma cells and prolong the survival of glioma-bearing mice [169]. Senescence (cell enlargement, flattening, increase of nuclei and nucleoli), followed by necrosis, accounted for CNF1-induced glioma cell death. A potential therapeutic feature of CNF1 for the most aggressive brain tumor, glioblastoma, seems possible because (i) glioma cells tend to diffuse profusely into adjacent healthy tissue and (ii) neural functions in the brain areas surrounding the tumor could be preserved due to the positive influence of CNF1 on learning, memory, and neuron plasticity [169]. In support, treatment with CNF1 reduced growth of glioblastoma at a symptomatic stage in a mouse glioma model [170]. Major advantages are that CNF1 acts rapidly, with high efficacy, and even a short exposure, i.e., a single administration, exerts a long-term effect as the active CNF1 appears to persist within the tissue for weeks [156,163,171].

Besides its potential use for neurological diseases and cancer, the CNFs may be useful for the treatment of other diseases. For instance, CNF-mediated influence on barrier function offers therapeutic options for skin fragility. A study by He et al. reported that CNF_Y_ was able to restore cell–cell adhesion in kindlin-2-deficient keratinocytes associated with abnormal cytoplasmic distribution of adherens junctions and desmosomal proteins [172]. Several studies also documented that the CNFs elicited adjuvanticity and are effective immunogens. This suggests the utility of this class of toxins as protective antigens and immunostimulants for prophylactic and therapeutic vaccinations [141,173,174,175,176]. In addition, CNF1 has been tested to prevent *Plasmodium falciparum* cytoadherence and induce the detachment of *P. falciparum*-parasitized erythrocytes (pRBC) from endothelial monolayers [177]. All these findings are powerful examples of possible biomedical applications of toxins.

As the actin cytoskeleton influences pain-related signaling, CNF1 was tested to assess whether it exhibits analgesic activity. In fact, peripheral and central administration of CNF1 counteracted formalin-induced inflammatory pain in mice [178]. The toxin was found to increase the expression of µ-opioid receptors (MORs), which probably induce the observed analgesic effects.

Protein toxins, such as the CNFs, are efficiently secreted into the environment, and they bind and trigger their uptake into host cells with a very high efficiency. Based on these properties, the non-toxic transport fragments of toxins can be engineered as carriers to deliver cargo (drug vehicles) into target cells for a variety of medical applications. A fusion of the receptor-binding domain of Shiga toxin and the translocation domain of exotoxin A has successfully delivered a cargo (N8A) to suppress the growth of hepatocellular HEp-G2 carcinoma cells [179]. The CNFs also have great potential as drug delivery tools. A study by Haywood et al. has successfully swapped the catalytic domains of CNF1, CNF2, CNF3, and CNF_Y_, indicating that the N-terminal protein of CNF_Y_ is the most powerful vehicle to deliver other proteins into host cells [26]. Our recent study further showed that fusions of the non-toxic N-terminal transport module of CNF_Y_ (D1–3) are able to transport larger cargo proteins, such as β-lactamase and GFP, into the cytoplasm of host cells, when fused to the C-terminal end of the D1–3 fragment [46]. As the CNFs target a variety of certain cell types, including immune cells, due to their receptor specificity, they could be used for the treatment of special cell subsets. This knowledge highlights that the CNFs have the potential for drug delivery into host cells. In future work, it will be important to refine this transport core region for efficient translocation to optimize CNF_Y_ as a drug delivery tool.

## 10. Conclusions and Perspectives

As we outlined in this review, CNF-like proteins are found in several bacterial genomes. They are implicated in bacterial virulence due to their cell-modulating functions through the activation of Rho GTPases. Presence of the toxins can be beneficial for the bacteria as they can enhance the initial colonization process. However, they can also be a disadvantage for long-term persistence and endurance in a commensal-like state as they can trigger host responses that enhance the elimination of the bacteria.

Despite the detrimental effects of the CNF family in their hosts, these toxins offer many properties that can be exploited for medical applications [157]. The detailed analysis of the structure and function of the toxins revealed that they are secreted in high amounts by the bacteria cell and promote efficient entry into a variety of host cells through a transport module that can be separated from its enzymatic functions and used as drug delivery tool. In addition, the versatile impact of CNFs on cell signaling processes and host functions can be exploited as therapeutic tools. The first attempts to use the toxins to treat neurological disorders and cancer in animal models have shown promising results and pave the way to possible therapeutic applications, in particular for severe diseases with no efficient therapy. Considering their pleiotropic effects on various cell processes, use of toxins for these purposes is certainly delicate, despite the fact that i.c.v. injections do not cause lethal effects in animals [157]. Moreover, many aspects of the toxins’ functions and their triggered virulence mechanisms, in particular their influence on the host immune response and inflammatory reactions, are still far from being understood and need to be clarified for their development as therapeutics.
